# Anatomic variations of the deltoid muscle insertion: a cadaveric study

**DOI:** 10.1016/j.jseint.2024.01.013

**Published:** 2024-02-17

**Authors:** Arjun Vohra, Benjamin Paul, Patrick Saunders, Youssef Galal, Stephen Yao, Clayton Hui, Evan Lederman, Michael McKee, Anup Shah

**Affiliations:** a University of Arizona College of Medicine-Phoenix, Banner University Medical Group, Phoenix, AZ, USA; b Creighton University School of Medicine, Phoenix, AZ, USA

**Keywords:** Deltoid, Insertion, Tuberosity, Anatomic variations, Cadaver, Shoulder

## Abstract

**Background:**

The deltoid is a trisegmented muscle with anterior, middle, and posterior components. While the clinical relevance of the presence of anatomic variations of the deltoid origin and insertion continues to be debated, the architecture of the deltoid muscle is more complex than initially believed. This study aimed to evaluate the gross anatomy of the deltoid muscle insertion by qualitatively and quantitatively characterizing the insertion and location of the deltoid muscle's anterior, middle, and posterior components. This information is valuable to surgeons as it raises awareness of potential variants that could be encountered during surgery, promotes mindfulness of neurovascular proximities, and reduces the likelihood of confusion between adjacent muscle fibers.

**Methods:**

Eight nonpaired, fresh-frozen clavicle-to-fingertip cadaveric shoulders were acquired for the study (6 left, 2 right). The average age of the cadavers was 79.5 years (range: 64-92). The standard deltopectoral approach was carried out on all specimens. The planes dividing the anterior, middle, and posterior deltoid were identified and marked. Once complete exposure had been achieved, digital calipers were used to record the size of the deltoid insertion. The specimens were qualitatively assessed to characterize the style of insertion they demonstrated.

**Results:**

The average length of the deltoid insertion was 39.45 ± 9.33 mm (n = 8). Six of the eight shoulders demonstrated an insertion style previously characterized in the literature. The remaining two shoulders highlighted an insertion pattern not previously described.

**Conclusion:**

The current study demonstrates a novel insertion pattern for the deltoid muscle that has not been previously characterized. This “step-off” insertion pattern shows that the anterior, middle, and posterior tendons are inserted superior-medial, directly on, and inferior-lateral to the deltoid tuberosity and was found in 2/8 of our cadaveric specimens.

The deltoid is the principal muscle responsible for shoulder abduction.^9^ This trisegmented muscle also significantly contributes to shoulder stabilization, preventing inferior displacement of the glenohumeral joint when the arm is adducted. The axillary nerve innervates the deltoid muscle via the ventral rami of C5 and C6. The muscle receives arterial supply from the deltoid and acromial branches of the thoracoacromial artery, the anterior and posterior humeral circumflex arteries, and the deltoid branch of the profunda brachii. The anterior head originates from the anterior acromion and the lateral third of the clavicle, the middle head from the lateral aspect of the acromion, and the posterior deltoid from the scapular spine.^9^ These three heads of the deltoid muscle are thought to converge and insert into the deltoid tuberosity on the lateral aspect of the humerus.^10^ The exact insertion orientation of the three deltoid heads has scarcely been described in the literature, and anatomic variations exist. A similar relationship is observed with the biceps brachii muscle, where the long and short muscle bellies converge into a single tendon and insert into the radial tuberosity. In addition, disruption of the distal tendon in either the deltoid or biceps brachii can potentially result in muscle weakness and contracture.^1^^,^^7^

However, the architecture of the deltoid muscle is more complex than initially described, as anatomic variations of the origin and insertion of the deltoid muscle have been described in the literature. Several case reports have found variations in the deltoid muscle belly and origin.^2^, ^3^, ^4^, ^5^ The deltoid insertion has been described in multiple ways. Morgan et al described the deltoid as a single insertion, whereas more recent studies have found the deltoid to be inserted in three discrete locations.^7^^,^^8^^,^^12^^,^^13^ The presence of anatomical variations of the deltoid insertion and the clinical relevance of such variations continue to be debated within the literature. These minor variations in insertion patterns can play a role in intraoperative considerations in various shoulder procedures, such as locking plate placement in surgical fixation of proximal humerus fractures.

This study aimed to further evaluate the gross anatomy of the deltoid muscle insertion by qualitatively and quantitatively characterizing the insertion and location of the deltoid muscle's anterior, middle, and posterior tendons. This information is valuable to surgeons as it raises awareness of potential variants that could be encountered during surgery, promotes mindfulness of neurovascular proximities, and reduces the likelihood of confusion between adjacent muscle fibers.

## Methods

**Figure 1:**
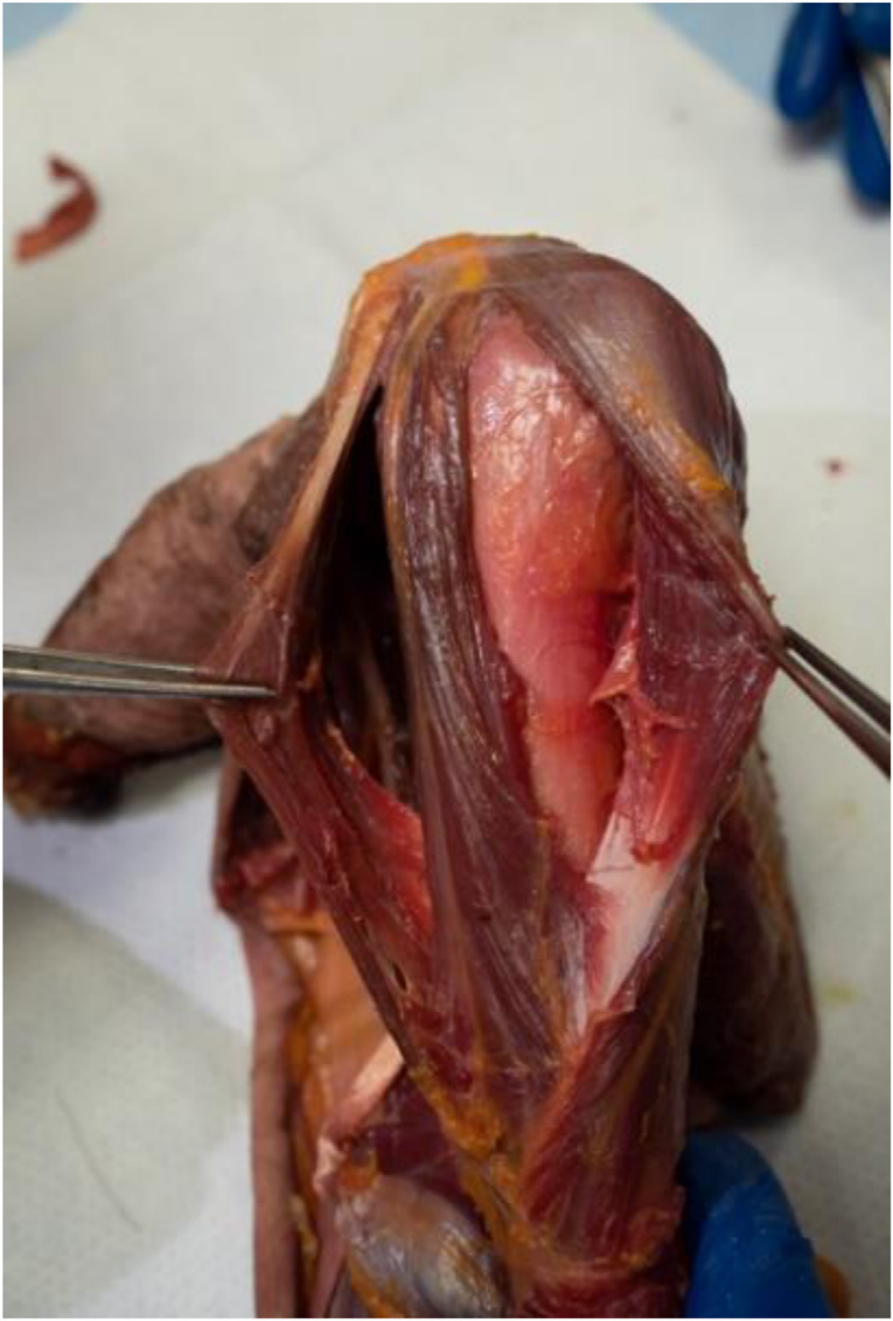
Deltoid insertion with dissected anterior, middle, and posterior planes.

**Figure 2:**
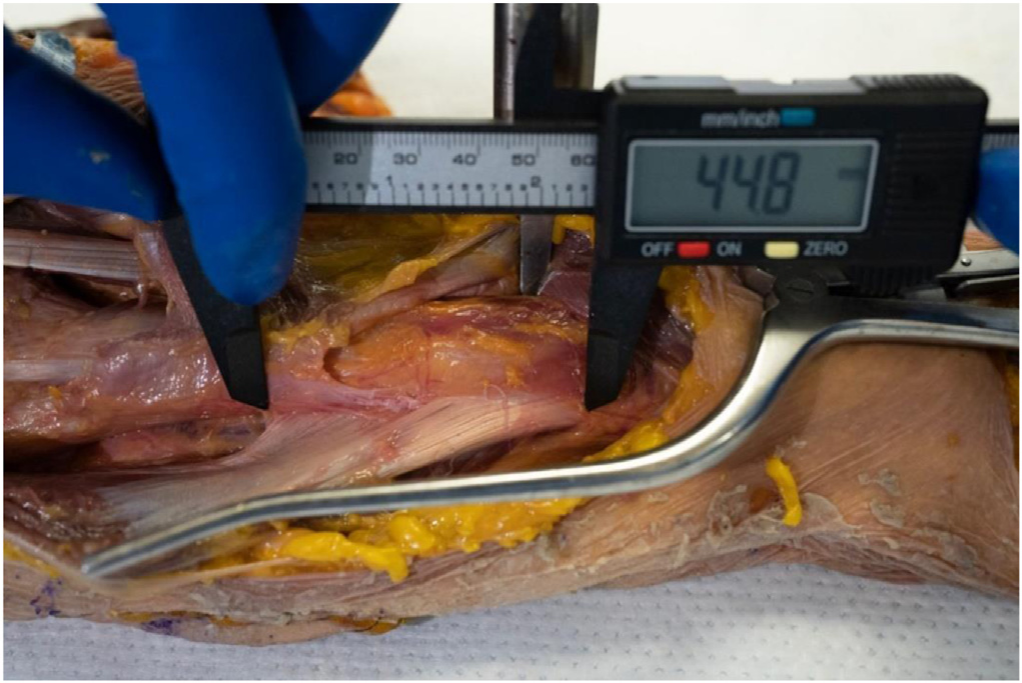
Deltoid muscle insertion length using a digital micro-caliper.

**Figure 3:**
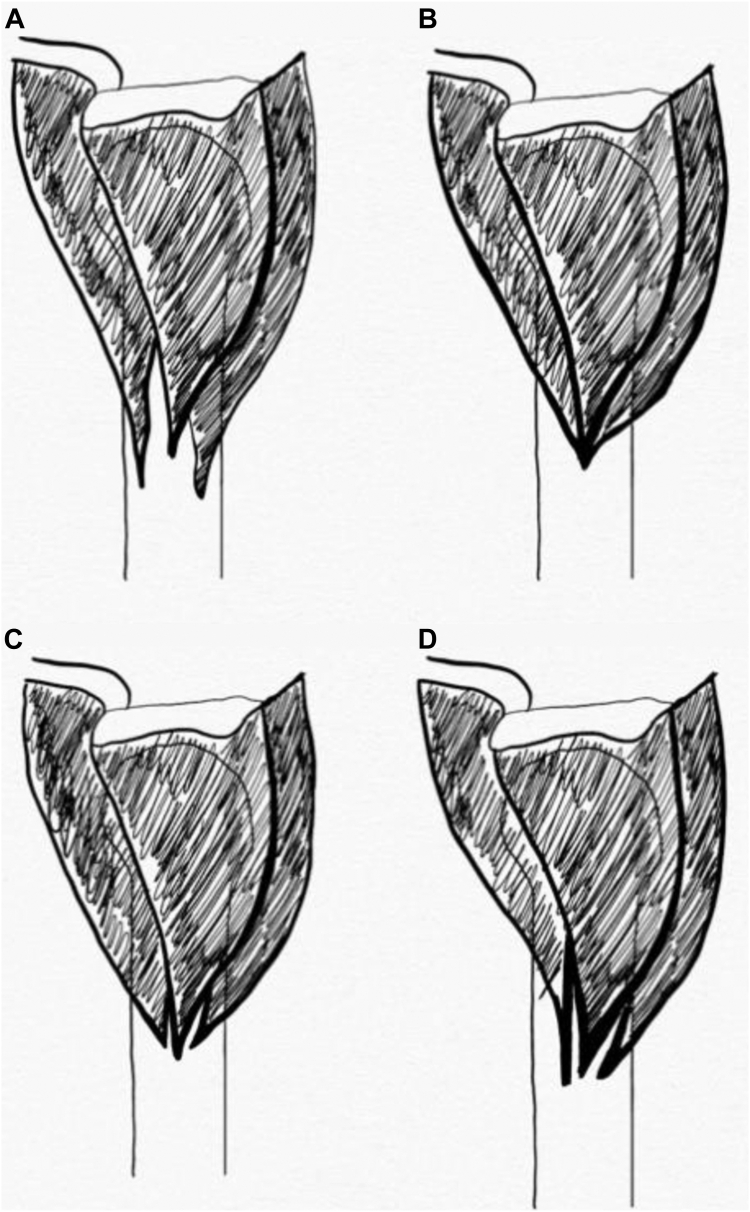
Anatomic variations of deltoid muscle insertion have been previously described in the literature. (**A**) Findings from Rispoli et al, (**B**) Findings from Morgan et al, (**C**) Findings from Klepps et al, (**D**) Findings from Sakoma et al.

Eight nonpaired, fresh-frozen clavicle-to-fingertip cadaveric shoulders were acquired for the purposes of the study (6 left, 2 right). A coding system was developed to identify individual specimens. Specimens were identified as L/R (for laterality), S (for shoulder), and followed by Specimen # (for example, left shoulder 1 was identified as LS1). The average age was 79.5 years old (64-92). One of the L-sided specimens (L3) had a prior history of malunion proximal to the deltoid insertion; however, the malunion did not affect the deltoid insertion, so it was not excluded from the study. The standard deltopectoral approach was used on all cadaveric specimens. The standard deltopectoral approach was carried out on all specimens. The deltoid muscle was delineated from its origin to its insertion, with initial care not to disturb the fascial interconnections of the muscle distally. The planes dividing the anterior, middle, and posterior deltoid were identified and marked. See Figure 1. The humerus was placed in a neutral position before measuring the deltoid insertion and characterizing the insertion pattern. This ensured a standardized degree of rotation before any measurements were made. Once exposure had been achieved, digital calipers were used to record the length of the deltoid insertion. See Figure 2. The specimens were qualitatively assessed to identify what style of insertion they demonstrated and were classified based on the insertion types demonstrated in the literature. See Figure 3. A single experienced investigator repeated the measurements and classifications for the entire study to ensure a consistent interpretation of the results.

## Results

The average length of the deltoid insertion was 39.45 ± 9.33 mm (n = 8). See Table I. The anterior, middle, and posterior deltoid insertion length was measured on the cadaveric specimen LS4 with lengths of 47.4, 47.9, and 45.2 mm, respectively.

**Table I:** Deltoid insertion lengths and insertion pattern classification.

Cadaveric specimen	Deltoid insertion length (mm)	Insertion pattern
LS1	42.4	Morgan et al^8^
LS2	25.3	Morgan et al^8^
LS3	38.1	Klepps et al^7^
LS4	46.9	Sakoma et al^13^
LS5	43.0	Step-off
LS6	47.8	Step-off
RS1	27.3	Morgan et al^8^
RS2	44.8	Sakoma et al^13^
Mean	39.45	
Standard deviation	9.33	

**Figure 4:**
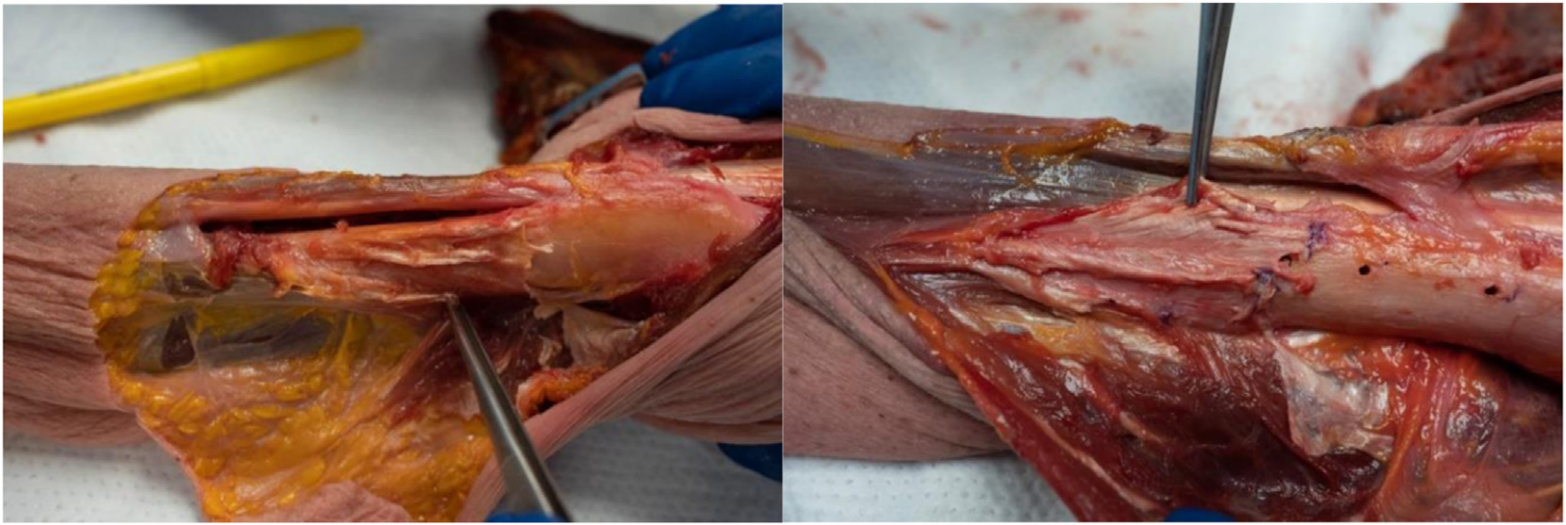
Two examples of different cadaveric specimens that display an anatomic variation of deltoid muscle insertion. Both figures display a diagonal step-off insertion pattern into the deltoid tuberosity where the anterior, middle, and posterior tendons are inserted superior-medial, directly on, and inferior-lateral to the deltoid tuberosity, respectively. The forceps in the *left* image are retracting the surrounding tissue to expose the step-off orientation of the deltoid insertion. The forceps in the *right* image are grasping the anterior deltoid muscle insertion to expose the step-off orientation.

The orientation and site of insertion of the anterior, middle, and posterior tendons of the deltoid muscle of each specimen were observed and classified based on the insertion patterns described in previous studies. Three of the eight shoulders followed the insertion pattern suggested by Morgan et al.^8^ Two out of eight specimens followed the insertion pattern described by Sakoma et al.^13^ One cadaver demonstrated the insertion pattern outlined by Klepps et al.^7^ The remaining two shoulders had an insertion pattern that followed a "step-off" pattern that was not previously described in the literature, see Figure 4. No shoulders followed the insertion style characterized by Rispoli et al.^12^

## Discussion

**Figure 5:**
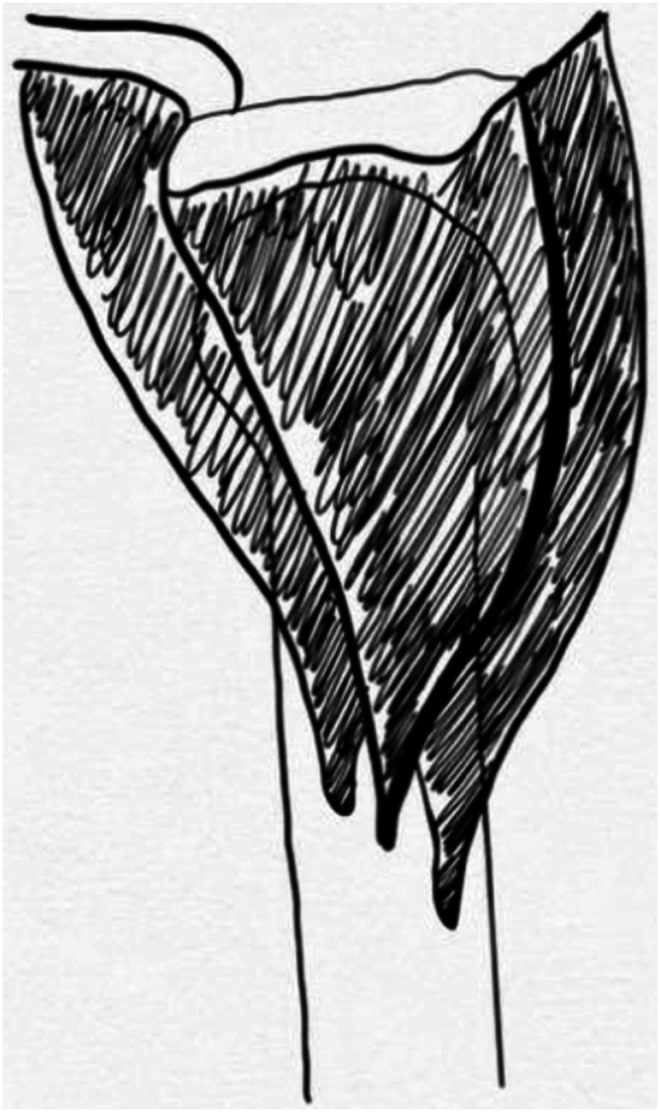
Illustrated example of the diagonal step-off insertion pattern in a *left* shoulder.

In the present study, the deltoid muscle's anterior, middle, and posterior tendons were inserted into the humerus in four different configurations, a finding consistent with the previous literature.^7^^,^^8^^,^^13^ The most commonly identified insertion style was the one indicated by Morgan et al.^8^ Additionally, two of the eight cadaveric specimens displayed an anatomic insertion pattern that has not been previously described in the literature. Figures 4 and 5 highlight this anatomic variation, displaying a “step-off” insertion pattern where the anterior, middle, and posterior tendons are inserted superior-medial, directly on, and inferior-lateral to the deltoid tuberosity, respectively. To our knowledge, this is the first time this variation has been identified, and it may be rather common, considering the pattern was present in one-fourth of the shoulders studied.

Awareness of the presence of anatomic variants for deltoid muscle insertion continues to grow. Klepps et al were the first to investigate the deltoid insertion pattern and described it as a V-shaped pattern with three discrete insertions.^7^ Klepps et al also observed the anterior and posterior tendons converging obliquely and the middle tendon passing vertically.^7^ The mean length of the anterior tendon was 112 mm, and the posterior tendon was 87 mm. The mean length of the anterior deltoid insertion was 69 mm anteriorly and 65 mm posteriorly. They characterized the posterior and middle deltoid as having broad tendinous bands, whereas the anterior deltoid is a long, thin band.^7^ Contrarily, Morgan et al later described the deltoid muscle as having a single, broad insertion.^8^ This was the most commonly identified insertion pattern in our study. A more recent study in 2009 by Rispoli et al found the deltoid to insert on the humerus in two to three macroscopically discernible locations, forming an arch-like or W-shaped configuration.^12^ In their study, the mean length of the anterior insertion was 70 mm, the middle was 48.4 mm, and the posterior was 63.4 mm. Although we did not identify any cadavers with this particular deltoid insertion pattern, our measurements of anterior, medial, and posterior deltoid insertion lengths were most similar to those reported by Rispoli et al.^12^ Sakoma et al described the deltoid muscle as having seven muscular segments that converge into three discrete insertions.^13^ They concluded that the anterior tendon was parallel to the humeral shaft, whereas the middle and posterior insertions were superoposterior against the humeral shaft. Two of our eight cadavers were consistent with the insertion pattern described by Sakoma et al^13^ Figure 3 highlights the anatomic variations of the deltoid muscle insertion previously described in the literature. While the orientation of the muscle fibers in the pattern described by Sakoma et al^13^ and Klepps et al^7^ are indeed similar, the entheses of the anterior, middle, and posterior tendons are the principal difference in these patterns. In addition, it is possible that internal or external rotation may impact the orientation of the fibers; however, the discrete tendinous orientation into the humeral shaft does not change. The present study focused on the anterior, middle, and posterior deltoid enthesis, irrespective of the orientation of the fibers that could be affected with shoulder rotation.

There are multiple procedures in which knowledge of the deltoid insertion and its insertion pattern variations is crucial. During open reduction and internal fixation of proximal humerus fractures, the deltoid muscle insertion may be compromised with plate fixation. Klepps et al concluded that partial release greater than 1/5th of the anterior deltoid insertion could result in weakness and contracture.^7^ Reverse total shoulder arthroplasty relies on the deltoid muscle for shoulder mobility and is an indicated procedure in the event of a failed internal fixation of a proximal humerus fracture. Thus, if the deltoid muscle has been compromised during open reduction and internal fixation of proximal humerus fractures, this may contribute to the inferior outcomes reported for patients who receive a reverse total shoulder arthroplasty after prior proximal humerus fracture fixation.^6^^,^^11^ Moreover, current advancements in plate technology, specifically the development of curvilinear locking plates, are designed to avoid deltoid insertion. Thus, surgeons should be wary of the potential deltoid insertion variations described in the present study and previous literature. Knowledge of this anatomy will enhance a surgeon's ability to successfully implant hardware without significantly compromising the deltoid muscle's form or function.

There are several limitations to this study. This study had a small sample size, with only eight cadaveric specimens used. In addition, the deltoid insertion length of the anterior, middle, and lateral heads was only directly measured on one specimen. Deltoid insertion pattern classification is partially subjective; however, only one experienced operator was allowed to classify the cadavers, decreasing the variability present. Future directions of this study should explore advancements in plate technology that would better preserve the deltoid muscle. Additionally, larger-scale cadaveric studies should be utilized to fully delineate the incidence of these various insertion patterns and aid surgeons in their intraoperative decision-making.

## Conclusion

The current study demonstrates a novel insertion pattern for the deltoid muscle that has not been previously characterized. This "step-off" insertion pattern shows that the anterior, middle, and posterior tendons are inserted superior-medial, directly on, and inferior-lateral to the deltoid tuberosity and was found in 2/8 of our cadaveric specimens.

## Disclaimers:

Funding: No funding was disclosed by the authors.

Conflicts of interest: Anup Shah is a consultant and receives royalties from Arthrex, Inc., Medacta USA, Inc., and ImpactOrtho, Inc. Michael McKee is a consultant and receives royalties from Stryker Corporation. Evan Lederman is a consultant and receives royalties from Arthrex, Inc. The other authors, their immediate families, and any research foundation with which they are affiliated have not received any financial payments or other benefits from any commercial entity related to the subject of this article.
